# Clumped Isotope Temperature Reconstruction Using Stalagmite Drip Cups

**DOI:** 10.1002/rcm.70027

**Published:** 2026-01-20

**Authors:** Stuart Umbo, Maria Box, Aviva Intveld, Jack Longman, Sevasti Modestou, Stacy A. Carolin, Daniel H. James, Alfredo Martínez‐García, Carlos Peraza Lope, Mark Brenner, David Hodell, Sebastian F. M. Breitenbach

**Affiliations:** ^1^ School of Geography and Natural Sciences Northumbria University Newcastle‐Upon‐Tyne UK; ^2^ Department of Natural Sciences Manchester Metropolitan University Manchester United Kingdom; ^3^ Department of Earth Sciences University of Cambridge Cambridge UK; ^4^ Department of Earth Sciences University of Oxford Oxford UK; ^5^ Institute of Archaeology University College London London UK; ^6^ Department of Climate Geochemistry Max‐Planck Institute for Chemistry Mainz Germany; ^7^ Instituto Nacional de Antropología e Historia Centro INAH Yucatán Mérida Mexico; ^8^ Department of Geological Sciences & Land Use and Environmental Change Institute University of Florida Gainesville Florida USA

**Keywords:** carbonates, clumped isotopes, drip cup, isotopic equilibrium, palaeoclimate, speleothem, temperature reconstruction

## Abstract

**Rationale:**

Application of clumped isotope palaeothermometry to speleothems (carbonate cave deposits, e.g., stalagmites and flowstones) has been restricted largely to subaqueous samples because of kinetic fractionation processes that occur during subaerial speleothem formation, which lead to erroneously high inferred temperatures. Speleothems are spatially near‐ubiquitous terrestrial archives that can be dated accurately over million‐year timescales. Thus, wider application of the clumped isotope technique in speleothems could dramatically increase our understanding of terrestrial thermal history. In this study, we assessed the potential of speleothem drip cups (concave depressions at a stalagmite apex in which dripwater accumulates to create a subaqueous environment) to yield reliable palaeotemperature inferences.

**Methods:**

We sampled along two isochronous layers that extend across both sides of a pronounced drip cup in stalagmite MAYA 22‐7 from Cenote Ch'en Mul, Yucatán, Mexico, which was dated to 1650 ce ± 23 years. We measured bulk stable (δ^18^O and δ^13^C) and clumped (Δ_47_) isotope values at increasing distances from the drip cup centre to test for kinetic fractionation effects.

**Results:**

Lower δ^18^O, δ^13^C, and higher Δ_47_ values were obtained from the drip cup's central subaqueous zone compared with the subaerial flanks, demonstrating reduced isotope fractionation in the subaqueous zone. Average clumped isotope temperatures (*T*_Δ47_) inferred from subaqueous drip cup samples are 1°C–2°C higher than modern cave temperatures and 3°C–7°C warmer than estimated formation paleotemperatures derived from nearby regional reconstructions and TEX_86_ analysis of our sample. This suggests a persistent degree of clumped isotope kinetic effects.

**Conclusions:**

Despite persistent kinetic effects, lower inferred temperatures from subaqueous drip cup samples suggest closer to equilibrium precipitation compared with subaerial samples. We propose that drip cup carbonates have the potential to yield reliable palaeotemperatures and describe a widely applicable test for clumped isotope kinetic effects in speleothem drip cups by sampling across isochronous layers.

## 1 Introduction

Clumped isotope analysis offers the potential for direct inference of palaeotemperature (i.e., formation temperature) from carbonate archives, through determination of the degree to which rare and heavy isotopes bond (or clump) together within the carbonate lattice [1, 2]. The extent of heavy–heavy isotope bonding is quantified as the Δ_47_ value—a measure of the ratio of mass 47 to mass 44 CO_2_ liberated when the carbonate is reacted with acid and normalised to the ratio expected given a stochastic distribution of isotopes within the lattice [3]. The Δ_47_ value exhibits an experimentally quantifiable relationship with the carbonate formation temperature [4], enabling palaeotemperature inference from a myriad of natural carbonate archives including, but not limited to, marine foraminifera [5], gastropods [6], ostracods [7, 8] and palaeosol carbonates [9]. A few studies have utilised speleothems (carbonate cave deposits, e.g., stalagmites and flowstones) for clumped isotope temperature reconstructions [10], yet widespread application remains largely unrealised because of frequent isotopic disequilibrium (kinetic) effects during speleothem deposition [11].

Speleothems form from the dissolution of limestone bedrock, which is transported to the cave environment in dripwaters. Since caves are typically low carbon dioxide environments compared with the overlying soils, CO_2_ degasses from the dripwaters upon entering the cave, causing oversaturation of the dripwater and precipitation of CaCO_3_. U‐series dating of speleothems yields exceptionally well dated records on million‐year timescales [12] across the terrestrial realm. Thus speleothem‐derived palaeotemperature estimates offer the potential to markedly expand our understanding of past terrestrial environments.

Emerging speleothem‐based temperature reconstruction techniques have achieved some success, but their widespread use is hindered by low concentrations of proxy variables or the requirement for specific depositional conditions during speleothem formation. For example, the stable isotope composition of ‘fossil water’ inclusions can be measured [13, 14]. These are derived from dripwaters, and thus, with knowledge of both calcite and dripwater isotopic composition, formation temperature can be calculated from experimentally determined water‐calcite oxygen isotope fractionation relationships [15]. Krüger et al. [16] utilised such fossil water inclusions to estimate paleotemperatures by laser excitation, and measurement of the subsequent liquid–vapor homogenisation temperature. Unfortunately, fluid inclusions are typically rare, and often absent from the speleothem fabric, limiting the number of samples to which the technique can be applied, thereby compromising the temporal resolution of the inferred palaeotemperature timeseries. In addition, the Krüger et al. methodology is limited to temperatures above approximately 10°C. Recent studies have applied the TEX_86_ palaeothermometer in speleothems to infer temperatures during the last interglacial [17, 18]. TEX_86_ estimates are based on the temperature dependence of the degree of cyclisation of Glycerol Dialkyl Glycerol Tetraethers (GDGTs), that is cell membrane lipids produced by archaea [19]. Whilst the TEX_86_ palaeothermometer is routinely applied to marine sediments [19], it remains relatively novel in speleothem studies [18, 20]. Successful application in speleothems requires sufficiently high biomarker concentrations, larger sample sizes (ca. 1 g), and the ability to infer low (< 5°C), and high (> 25°C), temperatures is hindered by calibration uncertainties [21]. Finally, Drysdale et al. (2020) [22] showed that magnesium concentration was correlated with temperature in subaqueous (precipitated beneath the water table) speleothems and proposed that it could be used to infer palaeotemperature directly. However, speleothems formed under such conditions are rare, precluding widespread application of the technique.

Clumped isotope geothermometry has been applied successfully to a limited number of subaqueous speleothems [23, 24]. Since all speleothems are technically subaqueous, we use the term ‘subaqueous’ throughout this study to refer specifically to samples forming below a body of water (e.g., cave pearls, pool rims, and drip cups) and ‘subaerial’ to refer to those forming under thin (typically < 0.6 mm) [25] water films (e.g., stalagmites and flowstones). In subaerial speleothems, rapid CO_2_ degassing from thin superficial water films on the carbonate surface frequently leads to preferential removal of light isotopes from the dissolved inorganic carbon pool (DIC = CO_2_(aq), ${\mathrm{HCO}}_{3}^{-}$, ${\mathrm{CO}}_{3}^{2-}$), leading to isotopic fractionation [26, 27]. This results in erroneously low Δ_47_ values and thus, inferred clumped isotope temperatures (*T*_Δ47_) that are too high [28]. The success of clumped isotope temperature reconstructions on subaqueous samples has been attributed to suppressed CO_2_ degassing (and therefore slower carbonate precipitation) compared with subaerial samples [28, 29], which enables sufficient time for full isotopic exchange between the DIC and precipitating carbonate. We propose that a minimum water depth must exist, under which isotopic equilibrium is achieved, given a certain DIC concentration and cave *p*CO_2_. Broadening the application of the clumped isotope technique to more speleothem samples would improve palaeotemperature inference and additionally provide reconstruction of the isotopic composition of speleothem drip waters—themselves a reflection of local hydrology.

We tested the application of the Δ_47_ thermometer to speleothem drip cups—concave stalagmite apexes in which dripwater accumulates to create a subaqueous environment for carbonate deposition. Since drip cups are relatively common features of speleothems, proof that the carbonate within them precipitates in isotopic equilibrium would substantially expand the range of speleothem samples from which palaeotemperatures could be derived. Where drip cups extend along the growth axis of a stalagmite, there exists the potential to reconstruct a temperature timeseries. Such records can be dated accurately with U‐series methods over million‐year timescales.

**FIGURE 1:**
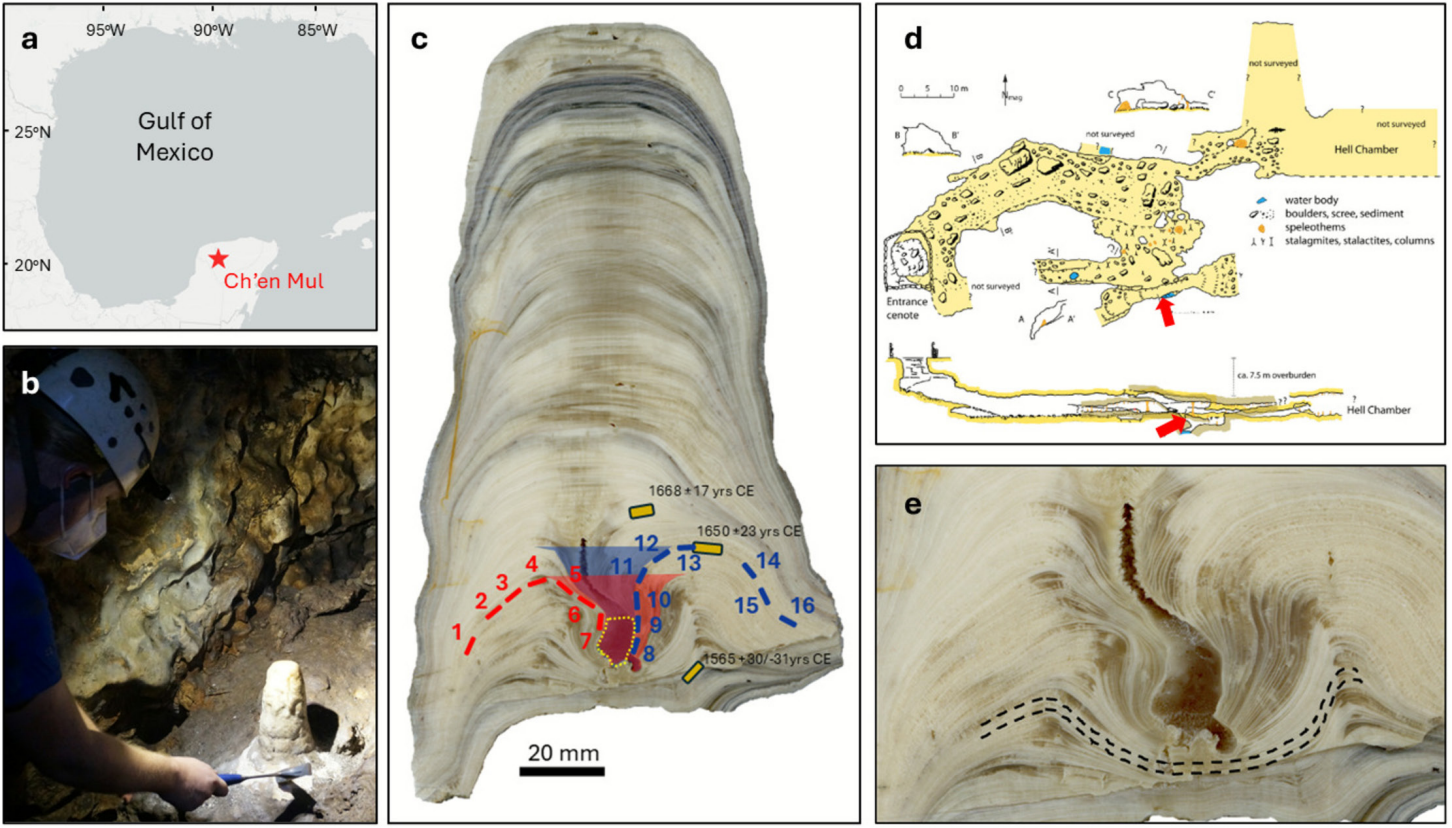
(a) Location of Ch'en Mul Cenote on the NW Yucatán Peninsula, Mexico. (b) Photograph of MAYA‐22‐7 in situ. (c) Cross section of stalagmite MAYA‐22‐7 showing dating and sampling locations (seven samples in red on the left side, nine in blue on the right). Shaded areas show the subaqueous zone where isotopic equilibrium precipitation is expected (red = left isochron, blue = right isochron). The yellow dashed region shows the TEX_86_ sampling location and yellow boxes U‐Th ages. (d) Cave map: plan and side view (from Homann et al., 2023 [30]). Red arrows show the location of Maya‐22‐7. (e) Cross section close‐up of the MAYA‐22‐7 drip cup, highlighting an example of a continuous growth layer between the left and right sides.

We hypothesise that pools within speleothem drip cups may be sufficiently deep to limit the rate of CO_2_ degassing relative to the volume of the DIC pool, thus providing suitable conditions for carbonate deposition in isotopic equilibrium. We tested this using stalagmite MAYA‐22‐7 from Cenote Ch'en Mul, Yucatán, Mexico, which has a deep drip cup in its lower section (Figure 1). We analysed Δ_47_ and stable isotope values (δ^13^C and δ^18^O) along two stalagmite growth intervals (isochronous layers), one on each side of the drip cup, to test for clumped and stable isotopic equilibrium, and assess the reliability of our Δ_47_ temperature inferences with comparison with an independent TEX_86_‐derived estimate. We propose that this approach can be used to test for clumped isotopic equilibrium in speleothems, in a similar manner to the conventional ‘Hendy test’, which is applied to test for equilibrium in stable isotopes [31].

## 2 Methods

### 2.1 Site and Sample Description

Stalagmite MAYA‐22‐7 was collected in August 2022 from Ch'en Mul Cenote in northern Yucatán, Mexico (N 20.63°, W 89.46°) (Figure 1a). A vertical sinkhole marks the entrance of the cenote, which then extends horizontally north‐eastward, directly underneath the Postclassic Maya archaeological ruins of Mayapán. A thin overburden of 7–8 m facilitates rapid infiltration of water, and sandy to clayey sediment covers the passage floor (Figure 1d). Approximately 100 m of the cave has been surveyed, beyond which it extends for an unknown distance [30]. Hourly monitoring between August 2022 and August 2023 yielded a mean air temperature of 25.7°C ± 0.6°C (2 s.d.) within the chamber where MAYA‐22‐7 formed [32]. Daily surface temperature and precipitation data are available from the GHCN weather station at Merida International Airport (N 20.98°, W 89.65°), ~43 km north of Mayapán. The mean surface air temperature between 1983 and 2023 was 26.5°C, with the warmest/coldest monthly mean temperatures of 29.1°C/23.3°C recorded in May/January. The highest/lowest mean monthly rainfall totals of 142.7 and 19.4 mm were recorded in June and January, respectively.

Stalagmite MAYA‐22‐7 was actively forming when it was collected from a narrow passageway in the south end of the cave (Figure 1b,d). Measuring 188 mm along its growth axis, it features a ~41‐mm‐deep (measured from the base to the growth apex) and ~45‐mm‐wide (measured between growth apexes [rims] on either side) drip cup near its base. Multiple cross‐sectional slices revealed that the MAYA‐22‐7 drip cup formed from a single drip site, rather than being two stalagmites that fused together. Furthermore, petrographic observations show that the growth layers are continuous across the drip cup, with thicker layers at the drip cup rim and thinner layers at the centre (Figure 1e). This indicates that the drip cup is a primary growth feature formed because of slower growth at the centre relative to the rim, rather than secondary dissolution of previously precipitated carbonate, which would have resulted in disrupted growth layers.

The drip cup was previously U‐Th dated on the mirror‐slab at the Department of Earth Sciences, University of Oxford, giving bottom and top formation dates of 1565 + 30/−31 and 1668 ± 17 years ce, respectively (Figure 1c) [33]. This suggests rapid growth of ca. 0.5 mm/year on the drip cup flanks. The right‐side isochron along which isotopic measurements were made was dated to 1650 ± 23 years ce [32]. Concordance of the right‐side isochron and the U‐Th dating pit across both slabs was confirmed using layer counting. We assume minimal to no growth axis movement in the plane orthogonal to the sampled surface, such that the U‐Th sample effectively records the same age at depth on both slabs.

### 2.2 Isotope Analysis

We sampled along two isochronous layers on either side of the drip cup, using a computer‐aided Sherline 5410 micromill (Figure 1c). Seven samples of ~8 mg were taken from the left side, and nine from the right. The clumped and stable isotope compositions of each sample were measured using a Nu Instruments Nu‐Perspective isotope ratio mass spectrometer, coupled to a NuCarb sample preparation system at Northumbria University's NICEST laboratory. Samples of mass 325 ± 25 μg were reacted at 70°C in concentrated phosphoric acid (*ρ* = 1.96 g L^−1^) for 30 min. Reactant gas was trapped in a liquid‐nitrogen‐cooled coldfinger, after which it was dehydrated at −80°C and passed over a 1‐cm cryotrap filled with Porapak absorbent material and cooled to −30°C to remove organic contaminants. Samples were measured in dual inlet micro‐volume mode over 40 sample/reference cycles, with a total integration time of 40 min. Long‐term instrument performance was monitored using a single measurement of standard IAEA‐C2, in each run, from which an external standard deviation of 0.023‰ across all measurement sessions was derived.

At least 15 replicate analyses were performed on each sample (Table 1). Contaminated samples and standards, identified by elevated Δ_48_ and Δ_49_^3^, were removed, and a drift correction, based on ETH‐3 δ^45^–δ^47^ was applied over each run, prior to pruning of ETH standard outliers (δ^45^, δ^46^, Δ_47_ > *x̄* ± 2*σ*). Clumped (Δ_47_) and stable isotope values were calculated using the D47crunch program in Python [36] inputted with the IUPAC parameters for ^17^O correction and isotopic ratios for VPDB and VSMOW [37, 38, 39] and ICDES Intercarb Δ_47_ values for ETH‐1, ETH‐2 and ETH‐3 from Bernasconi et al. [40]. Sample outlier pruning was performed (δ^13^C, δ^18^O, Δ_47_ > *x̄* ± 2*σ*) before calculating final isotope values. Clumped isotope (Δ_47_) uncertainty was calculated using the D47crunch package for Python, enabling full propagation of analytical uncertainty associated with standardisation to the ICDES reference frame [36].

**TABLE 1:** Stable and clumped isotope results and temperature inferences from MAYA‐22‐7.

	Position (L ➔ R)	Sample ID	Dist from centre (mm)	*N* (outliers)	δ^13^C (VPDB)	δ^13^C σ (VPDB)	δ^18^O (VPDB)	δ^18^O σ (VPDB)	Δ_47_ (ICDES)	Δ_47_ SE	D and V (2024)		Umbo et al. (2025)	
											*T*_Δ47_ (°C)	*T*_Δ47_ uncert (°C)	*T*_Δ47_ (°C)	T_Δ47_ uncert (°C)
Subaerial	1	MK22‐7‐15	−63	18 (3)	−4.89	0.02	−4.51	0.02	0.564	0.009	35.1	3.2	32.6	3.4
	2	MK22‐7‐5	−53	16 (2)	−5.02	0.01	−4.52	0.01	0.573	0.008	31.9	2.9	29.2	3.1
	3	MK22‐7‐1	−45	14 (3)	−6.15	0.01	−4.87	0.01	0.570	0.009	33.2	3.3	30.5	3.4
	4	MK22‐7‐2	−37	12 (3)	−6.34	0.02	−5.12	0.02	0.577	0.009	30.7	3.1	27.9	3.3
Subaqueous	5	MK22‐7‐3	−31	13 (3)	−6.31	0.02	−5.21	0.02	0.582	0.009	28.8	3.1	26.0	3.2
	6	MK22‐7‐4	−26	14 (2)	−7.00	0.02	−5.51	0.02	0.575	0.009	31.2	3.0	28.5	3.2
	7	MK22‐7‐12	−16	18 (3)	−7.16	0.04	−5.52	0.03	0.574	0.008	31.6	2.9	28.9	3.1
Subaqueous	8	MK22‐7‐13	10	17 (4)	−7.03	0.01	−5.51	0.02	0.574	0.009	31.7	3.0	29.0	3.2
	9	MK22‐7‐6	17	20 (3)	−6.78	0.02	−5.52	0.03	0.585	0.007	27.9	2.5	25.0	2.6
	10	MK22‐7‐7	24	22 (4)	−6.34	0.03	−5.40	0.02	0.572	0.007	32.4	2.6	29.8	2.7
	11	MK22‐7‐8	28	21 (4)	−6.20	0.02	−5.46	0.03	0.593	0.007	25.1	2.4	22.1	2.5
	12	MK22‐7‐9	35	21 (1)	−6.14	0.02	−5.32	0.02	0.574	0.007	31.7	2.6	29.0	2.7
Subaerial	13	MK22‐7‐10	41	16 (1)	−6.36	0.04	−5.23	0.02	0.574	0.008	31.5	2.8	28.8	3.0
	14	MK22‐7‐11	53	17 (2)	−6.14	0.02	−5.13	0.03	0.579	0.008	29.9	2.8	27.1	3.0
	15	MK22‐7‐16	61	18 (2)	−5.40	0.02	−4.86	0.03	0.563	0.008	35.6	3.0	33.0	3.2
	16	MK22‐7‐17	68	14 (3)	−5.34	0.04	−4.74	0.02	0.566	0.011	34.4	4.5	32.2	4.3

*Note: N* is the number of sample replicate analyses, with the number of outliers removed from final delta value calculations in parenthesis. *T*_Δ47_ was calculated with two calibrations, Daëron and Vermeesch (2024) [34] and Umbo et al., (2025) [35]. Data are presented left to right as seen in the cross section of the drip cup (Figure 1), with the grey horizontal bar indicating the centre.

### 2.3 GDGT (TEX_86_) Analysis

We obtained an independent palaeotemperature estimate for comparison to our clumped isotope reconstructions using the TEX_86_ index. The TEX_86_ index is based on the relative distribution of GDGTs with different numbers of cyclopentane rings. GDGTs were measured at the Organic Isotope Geochemistry Laboratory at the Max Plank Institute for Chemistry, according to the methods described in detail in Levy et al. (2023) [17]. Briefly, the speleothem sample was digested in HCl, and the organic fraction was extracted using dichloromethane and purified through a 5% deactivated silica column. Measurements were performed using an Agilent 1260 HPLC system coupled to an Agilent 6130 single‐quadrupole mass spectrometer [41].

### 2.4 Temperature Calibrations

We calculated clumped isotope temperatures (*T*_Δ47_) using the calibrations of Daëron and Vermeesch (2024) [34], a second‐order polynomial regression derived from biogenic and inorganic carbonates over a ~1000°C window, and Umbo et al. (2025) [35], a linear regression derived from 17 cave carbonates precipitated between 2°C and 76°C. Daëron and Vermeesch (2024) *T*_Δ47_ uncertainties were calculated using the D47calib program in Python. Uncertainties in *T*_Δ47_ derived with the Umbo et al. calibration were estimated following the methodology of Huntington et al. [42].

Our TEX_86_ temperature was calculated using the calibration of Baker et al. [20] (*T*_cave_ = −7.34 + 34.64 TEX_86_).

## 3 Results

### 3.1 Clumped and Stable Isotopes

**FIGURE 2:**
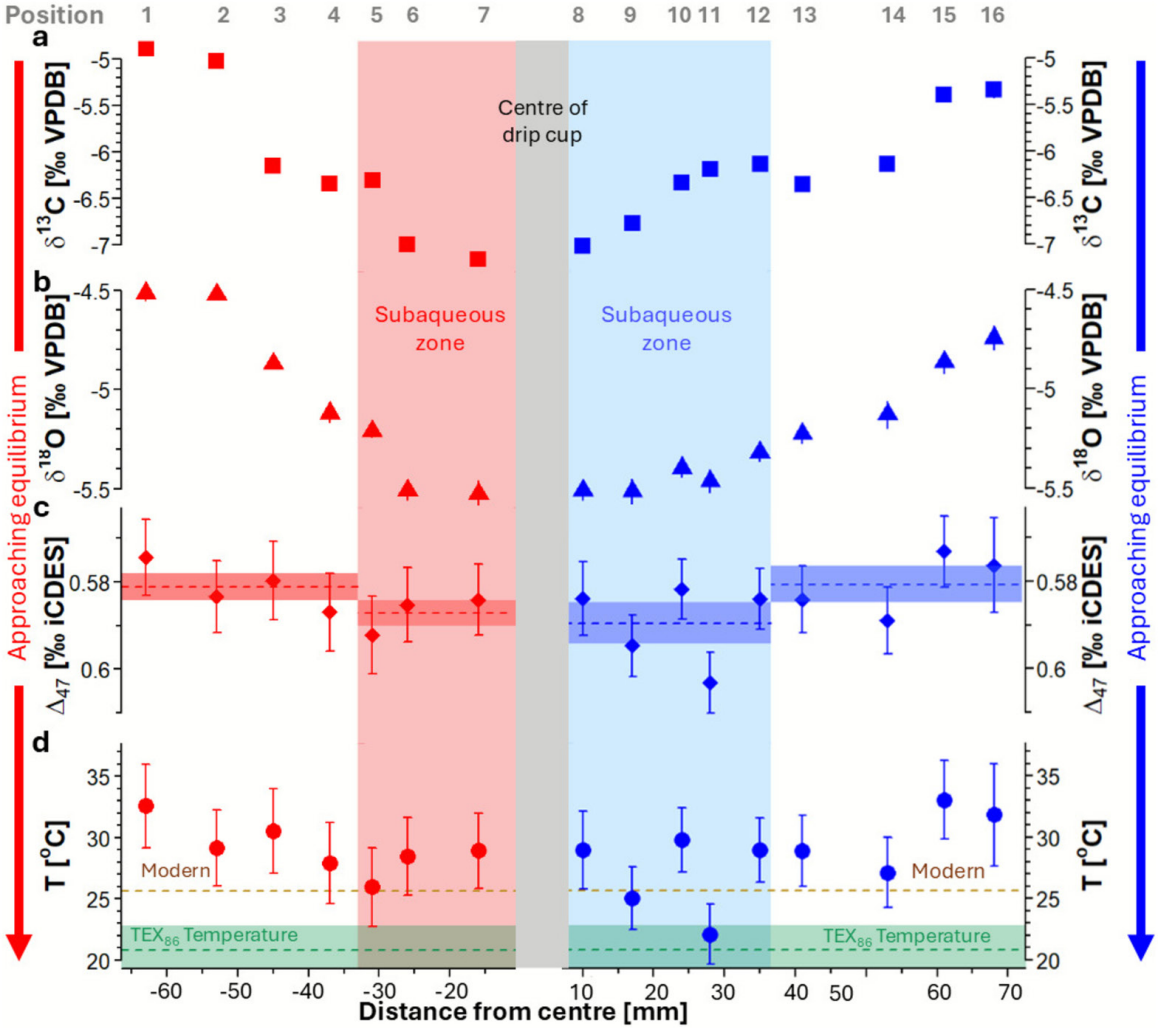
Isotope values and temperature inferences from speleothem MAYA‐22‐7 versus distance from the centre of the drip cup, measured along the two isochrons on either side of the drip cup: (a) δ^13^C (error bars are smaller than plot symbols); (b) δ^18^O; (c) clumped isotopes (Δ_47_), with average Δ_47_ for the subaerial and subaqueous zones shown by the horizontal dashed lines and the 75% confidence intervals by shaded bars; and (d) temperature inferences with modern and TEX_86_ temperatures shown by the horizontal brown and green dashed lines respectively, with the TEX_86_ uncertainty shown by the shaded green area. Arrows show predicted trends towards isotopic equilibrium. Stable isotope uncertainties are ± 1 standard deviation, and Δ_47_ uncertainties are ± 1 standard error.

Stable isotope compositions (δ^13^C and δ^18^O) are highest on the flanks (the outer edges of the drip cup) and decrease toward the centre (Table 1; Figure 2a,b). The decrease in stable isotope values is of greater magnitude on the left side of the drip cup (~2.1‰ in δ^13^C and ~1.0‰ in δ^18^O) than on the taller and steeper right side (~1.6‰ in δ^13^C and ~0.8‰ in δ^18^O).

Clumped isotope values (Δ_47_) increase toward the centre of the drip cup (Table 1; Figure 2c). On the left isochron, a minimum Δ_47_ value of 0.564 ± 0.009‰ ICDES was measured on the flanks of the drip cup and a maximum of 0.582 ± 0.009‰ ICDES (uncertainties are standard errors) in the subaqueous zone; however, all samples fall within standard error uncertainties. On the right isochron, a minimum Δ_47_ of 0.563‰ ± 0.008‰ ICDES was measured on the flanks of the drip cup and a maximum of 0.593‰ ± 0.007‰ ICDES was found in the subaqueous zone. Subaqueous samples on both isochrons are statistically higher than subaerial samples at the 75% confidence level (left isochron mean Δ_47(subaqueous)_ = 0.5772‰ ± 0.0029‰, mean Δ_47(subaerial)_ = 0.5711‰ ± 0.0031‰ and the right isochron mean Δ_47(subaqueous)_ = 0.5796‰ ± 0.0047‰, mean Δ_47(subaerial)_ = 0.5707‰ ± 0.0041 ‰; Table 2; Figure 2c).

**TABLE 2:** Clumped isotope summary data.

		N	Mean Δ_47_ (ICDES)	Δ_47_ confidence intervals (ICDES)			Mean T (°C)	T confidence intervals (°C)		
				± 95%	± 75%	± 68%		± 95%	± 75%	± 68%
Left side	Subaerial	4	0.5711	0.0052	0.0031	0.0027	30.1	2.0	1.2	0.8
	Subaqueous	3	0.5772	0.0049	0.0029	0.0025	27.8	1.8	1.0	0.9
Right side	Subaerial	5	0.5707	0.0070	0.0041	0.0036	30.3	2.7	1.6	1.4
	Subaqueous	4	0.5796	0.0080	0.0047	0.0041	27.0	2.9	1.7	1.5

Clumped isotope temperatures calculated with the Daëron and Vermeesch calibration are on average 2.7°C higher (range = 25.1°C–35.6°C, average = 31.3°C) than those calculated with the Umbo et al. approach (range = 22.1°C–33.0°C, average = 28.6°C). Hereafter, we limit our discussion to *T*_Δ47_ calculated using the Umbo et al. calibration, since this calibration is derived solely from cave carbonates precipitated at Earth surface temperatures

Both isochrons show a decreasing trend in *T*_Δ47_ towards the centre of the drip cup, with maximum reconstructed temperatures of 32.6°C ± 3.4°C and 33.0°C ± 3.2°C measured in the left and right subaerial zones, respectively, and minimum temperatures of 26.0°C ± 3.2°C and 22.1°C ± 2.5°C found in the left/right subaqueous zones. Subaerial samples produced statistically higher temperatures than subaqueous samples at the 75% confidence level (left isochron mean *T*_Δ47 (subaqueous)_ = 27.8°C ± 1.0°C, mean *T*_Δ47 (subaerial)_ = 30.1°C ± 1.2°C and the right isochron mean *T*_Δ47 (subaqueous)_ = 27.0°C ± 1.7°C, mean *T*_Δ47 (subaerial)_ = 30.3°C ± 1.6°C) (Table 2; Figure 2d).

### 3.2 TEX_86_

We obtained a TEX_86_ index of 0.813, giving rise to an inferred palaeotemperature of 20.8°C ± 2.0°C. The average analytical precision of the entire analytical procedure was ± 0.3°C, determined by multiple extractions of an internal speleothem standard. However, the uncertainty of the absolute cave temperature estimates from the empirical calibration is ± 2.0°C [20].

## 4 Discussion

### 4.1 Did the MAYA‐22‐7 Drip Cup Precipitate Near Isotopic Equilibrium?

CO_2_ degassing during speleothem formation drives kinetic fractionation by preferential removal of ^12^C and ^16^O from the DIC pool in speleothem‐precipitation waters, driving stable isotopic compositions of speleothem carbonate towards higher values [43]. Speleothem drip cups and cave pools, that is, subaqueous speleothem growth environments, likely make suitable targets for obtaining samples with reduced or even negligible fractionation effects because they have lower surface area to volume ratios compared with thin water films at stalagmite apexes and thus undergo comparatively lower CO_2_ degassing rates, given the relatively larger volume of the DIC reservoir.

The criteria for identification of stable isotopic equilibrium during the formation of stalagmite carbonate layers were described by Hendy [31]. The classic ‘Hendy test’ requires sampling along individual stalagmite growth (isochron) layers. A positive test, signifying kinetic fractionation during carbonate deposition, is indicated by increasing δ^13^C and δ^18^O values with increasing distance from the growth axis. This is caused by the preferential loss of light isotopes during CO_2_ degassing and potentially (for δ^18^O only) evaporation. In practice ‘true’ Hendy tests are near impossible to conduct because it is extremely difficult to sample along individual growth layers. However, the rapid growth in MAYA‐22‐7 enabled easier isochron sampling and we found a shift towards higher stable isotope values with distance from the centre of the drip cup, indicative of increasing kinetic fractionation processes (Figure 2, Table 1). The shift towards lower δ^13^C and δ^18^O towards the centre of the drip cup suggests increasingly ‘equilibrium‐like’ conditions with increasing water depth.

Controls on clumped isotopic equilibrium, however, differ from those on stable isotopes, and it is possible to achieve clumped isotope equilibrium despite kinetic processes acting on bulk stable isotope compositions (δ^18^O and δ^13^C). Watkins and Hunt [44] demonstrated with molecular modelling that stable isotope fractionation is largely controlled by mass‐dependent kinetics in the attachment/detachment of $\mathrm{HC}O_{3}^{-}$ and $CO_{3}^{2-}$ to and from the carbonate surface, whereas the clumped isotope composition of carbonates tended to be similar to that of the DIC pool when deposited at natural growth rates. This is a result of Δ_47_ values being calculated via normalisation to a stochastic distribution of oxygen and carbon isotopes with the same δ^18^O and δ^13^C as the measured sample and explains why natural carbonates such as foraminifera, with vastly different stable isotopic compositions, fall on the same temperature calibration line [4].

Clumped isotope disequilibrium is associated with lower Δ_47_ values in speleothems, leading to erroneously high inferred temperatures [26, 27]. Thus, a trend toward ‘near‐equilibrium’ conditions inside the drip cup should manifest as higher Δ_47_ values (conversely, increasing disequilibrium should give rise to lower Δ_47_ and higher temperatures toward the edges of the drip cup). In our example, we observed higher mean Δ_47_ values within the drip cup at the 75% confidence level. On the left side, the mean Δ_47subaqueous_ value is 0.5772 ± 0.0029 (*n* = 3), and the mean Δ_47subaerial_ value is 0.5711 ± 0.0031 (*n* = 4). On the right side, the mean Δ_47subaqueous_ value is 0.5796 ± 0.0047 (n = 4), and the mean Δ_47subaerial_ value is 0.5707 ± 0.0041 (*n* = 5) (Table 2). These results suggest that carbonate precipitation within the subaqueous section of the drip cup occurs closer to clumped isotope equilibrium than in the subaerial sections.

Whilst these findings suggest drip cups make promising targets for clumped isotope temperature reconstructions, our subaqueous clumped isotope temperatures (27.0°C–27.8°C) are ca. 6°C–7°C warmer than our TEX_86_ estimate (20.8°C ± 2.0°C) and ca. 3°C–4°C warmer than our estimate based on previous temperature reconstructions from the region. The sampled drip cup layer was dated to 1650 ± 23, during the Little Ice Age, a time when independent temperature reconstructions suggest a cooler than modern‐day regional climate. A sediment‐based reconstruction from the Sierra de Los Tuxtlas in southern Mexico suggests winter cooling of ~2°C during the Little Ice Age [45]. A similar magnitude of cooling is observed in marine records from the Gulf of Mexico [46]. Thus, based on previously published temperature reconstructions from the region, we estimate a cave temperature at the time of the drip cup formation of 25.7°C ± 0.6°C (modern monitored cave) −2°C = 23.7°C ± 0.6°C.

We cannot fully account for the warmer T_Δ47_ compared with our other palaeotemperature estimates, without some degree of clumped isotopic disequilibrium. Cave monitoring suggests temperature differences of up to 1°C in different parts of the cave [32] and it is possible that a different ventilation regime at the time of speleothem deposition may, in part, explain the warmer *T*_Δ47_ reconstructions. However, we deem it unlikely that ventilation changes explain the 3°C–7°C difference between our reconstructions and likely cooler‐than‐modern cave temperatures at the time of deposition. Thus, we suggest that some Δ_47_ disequilibrium pushed the MAYA‐22‐7 drip cup *T*_Δ47_ estimates to erroneously high values. Yet our relatively small offset is significantly less than the nearly +10°C offset previously inferred from subaerial Holocene and modern samples in caves in Western Germany [47], suggesting that although true equilibrium conditions in MAYA‐22‐7 may not have been achieved, kinetic effects were more limited than those previously observed in subaerial samples.

### 4.2 Wider Application of Clumped Isotopes in Speleothem Drip Cups

Despite being unable to unequivocally demonstrate clumped isotopic equilibrium within the drip cup of stalagmite MAYA‐22‐7, the increasingly ‘equilibrium‐like’ conditions towards the centre of the drip cup, indicated by lower stable oxygen and carbon isotope ratios, alongside higher Δ_47_ and thus reduced inferred mean *T*_Δ47_, suggest that speleothem drip cups are promising targets for clumped isotope temperature reconstructions.

Affek (2013) [48] demonstrated experimentally that clumped isotopic equilibrium between CO_2_ and water is achieved after ~10 h at 25°C. When rates of carbonate precipitation exceed rates of isotopic exchange between dissolved CO_2_ and water, there is insufficient time for DIC in solution to fully equilibrate [27]. Since precipitated CaCO_3_ largely inherits the clumped isotopic composition of the DIC pool [43], carbonate precipitated under such conditions exhibits clumped isotopic fractionation [23, 49]. Therefore, high speleothem growth rates, driven by rapid CO_2_ degassing, have been assumed to be the cause of kinetic fractionation in Δ_47_. In contrast, slow precipitation rates, associated with reduced degassing during subaqueous deposition, are thought to promote near‐equilibrium in δ^18^O, δ^13^C [50, 51] and Δ_47_ [24, 28].

However, the association between slow growth rate and Δ_47_ has been challenged in both laboratory [52] and naturally [53] precipitated carbonates, which showed no link between growth rate and Δ_47_ values. Kluge and Affek (2012) [47] suggested instead that the supersaturation state of precipitation waters was the dominant control on clumped isotope equilibrium. When carbonate‐precipitating waters are highly supersaturated with respect to carbonate, DIC dwell time within the solution is insufficient for full isotopic exchange and clumped isotope equilibrium is not achieved [47].

Supersaturation is modulated by cave environmental conditions and subaerial zone processes that were explored in detail by Kluge and Affek (2012) [46] and are summarised below. Upstream of the speleothem formation site, increased soil biological activity, that is, respiratory CO_2_ production, increases dripwater acidity and bedrock dissolution, ultimately leading to higher carbonate supersaturation within dripwaters. The opposite is true of prior carbonate precipitation (PCP—precipitation of carbonate upstream of the speleothem formation site), which reduces DIC concentration within final precipitation waters. The dominant PCP control on speleothem disequilibrium has also been demonstrated in theoretical models by Guo and Zhou [49]. Within the cave, *p*CO_2_ controls the rates of CO_2_ degassing, with greater rates associated with a higher concentration gradient between cave air *p*CO_2_ and dripwater DIC. Thus, a well‐ventilated cave with low *p*CO_2_ would be expected to increase the supersaturation state of precipitating waters and promote disequilibrium. Finally, low supersaturation is promoted when the ratio of water to DIC recharge is high, promoting mixing between DIC and water—that is, when drip rates are high.

Modern conditions at the MAYA‐22‐7 formation site suggest that kinetic effects likely impacted precipitation of our drip cup. The stalagmite was collected from near the cave entrance where ventilation would likely have been greatest. In addition, the thin overburden at Ch'en Mul would likely have led to low PCP rates, in turn leading to high supersaturation. This may explain the observed kinetic processes in the MAYA‐22‐7 stable isotope values, and the fact that we cannot rule out such processes in the clumped isotopic composition. However, increasingly ‘equilibrium‐like’ conditions toward the centre of the drip cup show the potential for equilibrium precipitation in speleothem drip cups under the more favourable conditions outlined above (low soil productivity and cave ventilation, high PCP and drip rates) which promote a lower supersaturation state. The lower surface area to volume ratio of cave pools and drip cup reservoirs (i.e., subaqueous speleothem growth environments), compared with thin water films, results in comparatively low CO_2_ degassing rates given the available volume of the DIC reservoir, leading to relatively lower pH and retaining the carbonate in solution. This suggests that speleothem drip cups represent valuable targets for clumped‐isotope‐based temperature reconstructions, and we encourage further exploration of this possibility using drip cups with different geometries and caves with different ambient conditions.

### 4.3 A Widely Applicable Test for Clumped Isotope Equilibrium in Speleothem Drip Cups

We demonstrated the application of a simple test for clumped isotopic equilibrium during speleothem formation, analogous to the conventional ‘Hendy test’, which is applied routinely to test for kinetic processes in stable isotope studies. By sampling along isochronous layers, clumped isotope kinetic processes can be identified by a shift to lower Δ_47_ values as kinetic fractionation influences increase. An observed plateau in Δ_47_ can be used to identify clumped isotope near‐equilibrium.

Drip cup morphologies are relatively common, and if it can be shown that the carbonate in them precipitated near equilibrium, they offer the potential to dramatically expand the number of speleothem‐based clumped isotope temperature reconstructions. Absolute temperature timeseries may even be derived where drip cup morphologies extend along the growth axes.

The recent development of dual clumped isotope analysis offers an alternative avenue to test for clumped isotopic equilibrium [54, 55]. Deviations from a theoretical equilibrium line in Δ_47_ vs. Δ_48_ space help identify kinetic processes that can be corrected for by projection of measured clumped isotope values to the equilibrium line along predicted kinetic trajectories [56]. However, dual clumped isotope measurements require instrument precision currently beyond the capabilities of most laboratories, and large sample sizes (> ~ 10 mg), which limits the spatial resolution of temperature reconstructions. Until dual clumped isotope measurements become possible with much smaller sample sizes, we propose our method as a feasible test for clumped isotope equilibrium deposition in speleothem drip cups.

## 5 Conclusions

We have provided evidence that clumped isotope values in carbonate samples from subaqueous areas of speleothem drip cups are closer to equilibrium than carbonate samples taken from subaerial carbonate formed along the same growth interval. Clumped isotope values from the two sites differ from one another statistically at the 75% confidence level. This is indicated by higher Δ_47_/lower reconstructed *T*_Δ47_ from subaqueous samples within the drip cup compared with those on the apex and flanks. Our findings suggest speleothem drip cups make good targets for reliable, accurately dated, clumped isotope temperature reconstructions.

Furthermore, we propose that measurement of Δ_47_ along isochron layers can be used as a means to test for clumped isotopic equilibrium precipitation in speleothems, analogous to the conventional ‘Hendy test’, used to test for stable isotope equilibrium deposition. We recommend wider application of this test to speleothem drip cups to expand the range of speleothem samples from which temperature reconstructions can be made.

## Author Contributions

SU oversaw writing of the manuscript and figure preparation. SB, DJ, CPL, MBr, and DH were responsible for sample collection. SB conceptualised the study. SU, SB, and JL milled samples. SU, SM, JL, and MBo performed clumped isotope analysis. AI and SC performed radiometric dating. AM‐G performed TEX_86_ analysis. All authors contributed to the review and editing of the manuscript.

## Conflicts of Interest

The authors declare no conflicts of interest.

## Data Availability

All datasets are available upon request from the corresponding author (stuart.umbo@northumbria.ac.uk) and will be made available on the Earthchem Archive database pending publication of the manuscript.
